# New Drugs, Appliances, and Things Medical

**Published:** 1890-06-14

**Authors:** 


### NEW DRUGS, APPLIANCES, AND THINGS MEDICAL.

[All preparations, appliances, novelties, etc., of which a notice is desired, should be sent to The Editor, The Lodge, Porchester Square, W.]

### RIZINE.

The Rizine Food Company have sent us samples of their manufacture. We have tested these and find them to be a very useful and digestible form of carbohydrate food. There are several interesting points about Rizine which are worth noting. It is already cooked. The rice is soaked, then treated by superheated steam and finally rolled, so that the ultimate product is a flake. This is one of the forms in which Rizine is sold, the other is merely the flakes ground down to a meal. By this cooking process, the starch granules are split up and probably partly started on the soluble or sugar-making track. This makes the food an easily digested one, as the saliva and pancreatic ferments have less to do than when acting on imperfectly cooked starch. Again, being cooked, Rizine requires less culinary treatment than the ordinary forms of cereal products; this makes it pre-eminently the material for dishes which require to be quickly made. We note that Rizine itself untreated in any way has a pleasant taste and feeling in the mouth. This, though apparently a rough test, is one of the most valuable for starchy foods. The slightest musty or disagreeable flavour can thus be detected. The product is also pleasant to the eye, and forms a variety of food which is not only nutritious but palatable as well. The company send out a capital little book of receipts for the use of Rizine in many combinations.

### THE PATENT STEEL WIRE DOMINION SPRING MATTRESS AND THE "LAWSON TAIT" BEDSTEAD.

We recently inspected at 58, Holborn Viaduct, the above. The Lawson Tait Bedstead is an adaptation of the Dominion Spring Mattress to the various forms of iron and brass bedsteads. The mattress is made of V's or links of steel coppered wire, hinged or looped together. At the two ends of the arrangement are a series of steel springs. Each of these is tested up to 80 lbs., and as there are from 60—80 of these in an ordinary-sized mattress, the corresponding elastic power is very great. The ends of the frames are made in an arch, so that pressure tends to equalize itself wherever it may be applied. This prevents jagging and unevenness, which two things often are met with in a wire-wove mattress. Again, as the links are in V's the pressure is distributed in a series of diagonal lines; this eases the stress on the supporting ends of the mattress. The meshes of the V's being large, there are less points of contact tending to wear out the superincumbent bedding and more space to ventilate it. We made careful trials of the mattresses and found them to be most comfortable. Mr. Adlam informs us that he is selling them in largely increasing numbers, and that many large hospitals and public institutions are replacing their old-fashioned beds by these. Among the most valuable qualities is that of requiring little or no repair. The steel wires and springs are coppered, so there is no rust—a great desideratum, especially in hospital beds. Again the absence of unevenness which requires periodically tightening up, makes the care of these bedsteads a light one in large institutions. Finally, we can heartily recommend these inventions, and hope the introducers may meet with an extended success.

### BENGER'S PREPARATIONS.

Messrs. Mottershead and Co., have forwarded to us samples of the above. The Liquor Pepticus and Liquor Pancreaticus are such old and valued friends that we have difficulty in finding fresh terms of praise for them, suffice it to say, that on making experimental trial of the specimens before us, we find them as good and active as ever. The firm have also put up their beef jelly and chicken jelly (both peptonized) in neat glass jars, thus doing away with any chance of metallic contamination. This, we believe to be a distinct advance, as the peptonized forms of meat preparations containing free acid, or alkali, would be likely to dissolve lead or other metals which are used in soldering the tins.

These jellies are not only useful in cases requiring predigested food, but are free from that unpleasant taste that partially digested food often has. The chicken jelly has the well-known flavour of chicken—"hen broth" our ancestors in the South of England called it. It makes an agreeable change from beef-tea or other broths. Change in flavour often means a meal enjoyed and digested by the patient, and so health and strength gained. Besides the above there is the well-known Benger's Food, most useful for dividing the curd of milk in the stomach, and so preventing the formation of large lumpy masses of practically insoluble casein. Messrs. Mottershead and Co., also put up the various digestive ferments in the forms of powders (pulvis pancreaticus alkalinus Benger), and pills of pepsin.

There are, besides, pancreatized oat flour and pancreatized lentil flour, both useful preparations, which can be alternated with the "food" when a change of diet is required. For the kitchen this firm manufactures a very pure and powerful essence of rennet for making fresh whey and junket.

### INGER'S BOOK-HOLDER.

Mr. Lucien Inger, of Holborn Viaduct, has sent us an ingenious invention of his called Inger's Patent Adjustable Book-holder. The size we have received will only take a small book of three hundred pages, and we have no information as to whether this patent book-holder is made in other sizes too. As it is it would no doubt prove a comfort to many readers, and the idea affords a wide field for development in various directions. No doubt for garden and seaside purposes, Inger's Patent] Adjustable Book-holder will become a popular and useful companion to students as well as to readers of lighter literature.

